# Supramolecular amplification of amyloid self-assembly by iodination

**DOI:** 10.1038/ncomms8574

**Published:** 2015-06-30

**Authors:** Arianna Bertolani, Lisa Pirrie, Loic Stefan, Nikolay Houbenov, Johannes S. Haataja, Luca Catalano, Giancarlo Terraneo, Gabriele Giancane, Ludovico Valli, Roberto Milani, Olli Ikkala, Giuseppe Resnati, Pierangelo Metrangolo

**Affiliations:** 1 Laboratory of Nanostructured Fluorinated Materials (NFMLab), Department of Chemistry, Materials, and Chemical Engineering ‘Giulio Natta’, Politecnico di Milano, Via Mancinelli 7, Milano I-20131, Italy; 2 VTT-Technical Research Centre of Finland, PO Box 1000, Espoo FI-02044, Finland; 3 Department of Applied Physics, Aalto University, PO Box 15100, Espoo FI-02150, Finland; 4 Dipartimento Beni Culturali, Università del Salento, Lecce I-73100, Italy

## Abstract

Amyloid supramolecular assemblies have found widespread exploitation as ordered nanomaterials in a range of applications from materials science to biotechnology. New strategies are, however, required for understanding and promoting mature fibril formation from simple monomer motifs through easy and scalable processes. Noncovalent interactions are key to forming and holding the amyloid structure together. On the other hand, the halogen bond has never been used purposefully to achieve control over amyloid self-assembly. Here we show that single atom replacement of hydrogen with iodine, a halogen-bond donor, in the human calcitonin-derived amyloidogenic fragment DFNKF results in a super-gelator peptide, which forms a strong and shape-persistent hydrogel at 30-fold lower concentration than the wild-type pentapeptide. This is remarkable for such a modest perturbation in structure. Iodination of aromatic amino acids may thus develop as a general strategy for the design of new hydrogels from unprotected peptides and without using organic solvents.

## Abstract (web summary)

Amyloid assemblies lie at the heart of many physiological functions, as well as being the cause of numerous diseases. Here, the authors subtly modify wild-type pentapeptides with halides, and discover that the new halogen bonding interactions have a remarkable influence on their physical properties.

## Graphical abstract

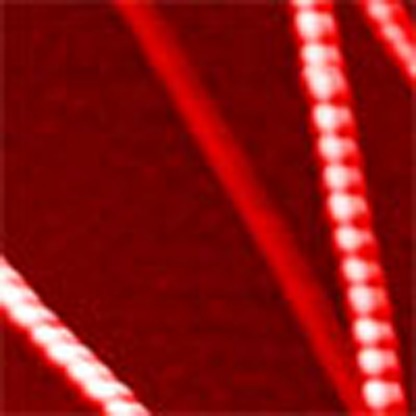

Amyloid fibrils are a class of nanomaterials that can be assembled from a wide variety of peptides and proteins and have an array of functional and pathological roles in nature^1^. In the latter, the self-assembly process can be triggered by peptide mutations and leads to the formation of insoluble fibrillar aggregates that are associated with Alzheimer’s, Parkinson’s, Creutzfeldt–Jakob and Huntington’s diseases^2^,^3^,^4^,^5^,^6^. Amyloid fibrils also perform a number of physiological roles including being constituents of protective envelopes of fish and insect eggs, essential amphipathic materials of fungi and bacteria and a vital constituent of spider silk^7^. This wide variety of physiological functions of amyloids has inspired many nanocomposites thanks to the robustness of these fibrillar materials, which are comparable to dragline silk and collagen, as well as biomaterial applications like tissue engineering^8^,^9^,^10^,^11^. In this context, it is important that the amyloidogenic peptide/protein self-assembles in a tunable and controlled manner to provide fibrils of well-defined architecture^12^.

Amyloid fibrils are characterized by their cross-β-sheet structures whereby bundles of β-strands form highly ordered filaments that run perpendicular to the fibre axis^11^,^13^. The stability of amyloid structures can be attributed to noncovalent interactions, namely hydrogen bonds, π–π stacking and hydrophobic interactions occurring between both side chain and backbone atoms, which hold the β-sheets and strands together^11^. On the basis of the assumption that noncovalent interactions are key to the formation of the amyloid structure, and since halogen bonds have been recently appreciated as new tools for supramolecular engineering^14^, we decided to study the impact of introducing halogen-bond donor groups, for example, iodine atoms, into an already known amyloidogenic motif to study the potential of halogen bonding in controlling and promoting amyloid self-assembly.

Although many modifications of amyloidogenic sequences have been utilized to tune their self-assembly behaviour^12^, halogenation has rarely been pursued and only on single amino acids or on a dipeptide^15^,^16^,^17^. The advantage of a strategy based on the introduction of halogen atoms on amyloidogenic motifs lies in the fact that halogenation is a minimal structural modification, which, on the other hand, may induce a large difference in the peptide supramolecular behaviour as a consequence of the rich variety of noncovalent interactions given by halogen atoms^18^. It has been shown previously that incorporation of single halogen substituents on the aromatic side chain of amino acids enhances their self-assembly into amyloid-like fibrils that promote hydrogelation in aqueous solvents^19^. In particular, fluorinated amino acids were found to function better than chlorinated and brominated analogues in terms of assembly kinetics and rigidity of the obtained hydrogels. This has been explained by the significant electronic effects of fluorine and the subsequent perturbation of the energetics of the π–π stacking interactions, although hydrophobic and steric effects could not be discounted^20^. Conversely, there is only one report on the fibrillar nanostructures given by a diiodinated dipeptide^17^ but, to the best of our knowledge, no detailed study on the effect of iodination on promoting/inhibiting the self-assembly of amyloidogenic peptides. Under the hypothesis that introducing an iodine atom on the *p*-position of the benzyl side chain of an aromatic amino acid may result in the formation of halogen bonding thus promoting fibrils’ formation, we studied the structure of *p*-iodo-phenylalanine (*p*-I-Phe) and the self-assembly behaviour of a series of iodinated derivatives of a known amyloidogenic core sequence.

## Results

### Single crystal X-ray structure of *p*-iodo-phenylalanine

The halogen bond^21^ is the strong, specific and directional interaction involving halogen atoms as electrophilic species^22^. Increasing in strength with halogen atom polarizability, halogen bonds are most efficient with iodine substituents. This rather new interaction is virtually unexplored in the field of amino acid and peptide self-assembly. For this reason, to evaluate the possibility of exploiting the halogen bond in the self-assembly of amyloid peptides/proteins, we have first studied the single crystal X-ray structure of the amino acid *p*-I-Phe.

**Figure 1:**
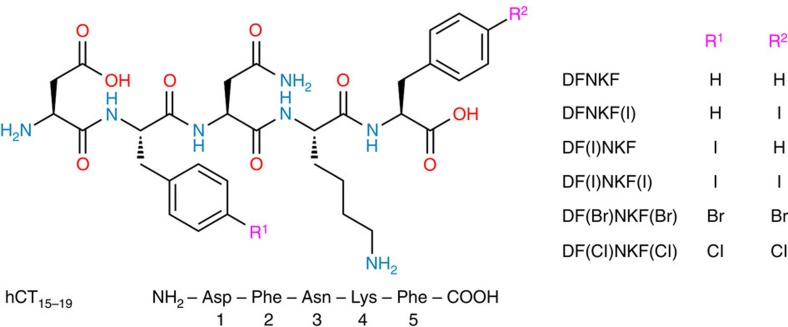
Molecular formulae of the peptides used in this study. The natural peptide DFNKF was modified by halogenation on the phenylalanine residues in the para position. Modifications are shown in the peptide sequence in brackets where F(I) denotes 4-iodophenylalanine, F(Br) denotes 4-bromophenylalanine and F(Cl) denotes 4-chlorophenylalanine.

The crystal structure of *p*-I-Phe (Supplementary Fig. 1 and Supplementary Table 1) highlights the amphoteric behaviour of the iodine atom, which functions as Lewis acid along the extension of the C–I bond and as Lewis base at the belt perpendicular to it (Supplementary Fig. 2a). In fact, the crystal packing of this iodinated amino acid is characterized by two strands of amino acids running along the *b* crystallographic axis and connected through hydrogen bonds (Supplementary Fig. 2b). These strands are then laterally assembled into two-dimensional supramolecular sheets thanks to type II iodine···iodine contacts^23^, that is, halogen bonds (I···I distance 3.7517(4) Å, I···I···I angle 89.66°), occurring at the strand surface (Supplementary Fig. 2c). Importantly, no sign of π–π stacking is observed, while C–H···π interactions involving the benzyl hydrogens contribute to aromatic side-chain ordering. Despite the fact that inferring structural information on the amyloid state from high-resolution X-ray diffraction analysis of small-molecule models is difficult, we introduced the *p*-I-Phe residue into the amyloidogenic core sequence DFNKF^24^ (NH_2_-Asp-Phe-Asn-Lys-Phe-COOH, Fig. 1), with the purpose of exploiting similar halogen bonds at the fibril strand surface to promote fibril–fibril lamination effects.

DFNKF is a short segment (residues 15–19) of the human calcitonin (hCT) hormone^25^, an amyloidogenic protein with 32 amino acids, whose amyloid formation is related to medullary carcinoma of the thyroid^24^. This particular pentapeptide is widely used for investigating the nanoscopic arrangement of a fibril complex since DFNKF forms well-ordered fibrils similar to the aggregates of hCT^24^,^25^. For this reason, we selected DFNKF as a simple model compound for fibril formation and obtained its halogenated derivatives by introducing I, Br and Cl atoms at the *p*-position of the phenylalanine (Phe) benzene ring. To study the effect on fibrillation of the introduction of halogen atoms on specific positions of the pentapeptide, we studied the 5-*p*-iodo-Phe derivative DFNKF(I), the 2-*p*-iodo-Phe derivative DF(I)NKF, the 2,5-bis-*p*-iodo-Phe derivative DF(I)NKF(I), as well as its bis-brominated and bis-chlorinated analogues DF(Br)NKF(Br) and DF(Cl)NKF(Cl), respectively (Fig. 1). Importantly, all of the studied peptides carry free amino (N) and carboxyl (C) termini.

### Hydrogelation properties of halogenated DFNKF derivatives

**Figure 2:**
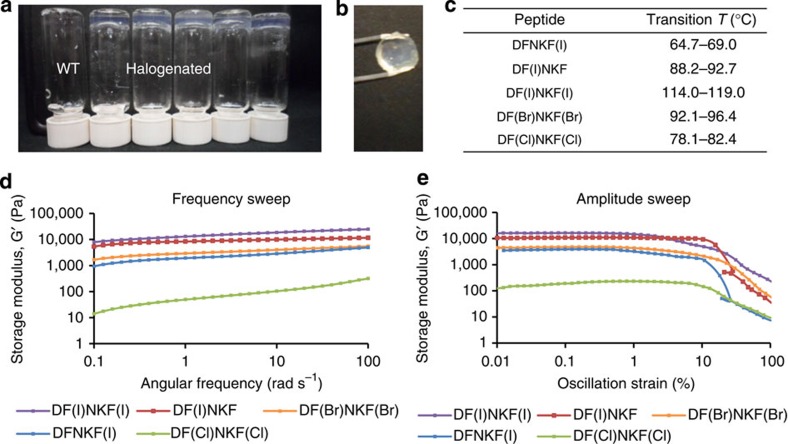
Characterization of hydrogels formed by halogenated DFNKF derivatives (**a**) Photograph of vials containing 15 mM peptide solutions upon aging 12 h at RT After this period, all halogenated peptides formed clear hydrogels whereas the wild-type peptide DFNKF (WT) remained as a viscous fluid. (**b**) The hydrogel formed by DF(I)NKF(I) was the most shape-persistent of all gels. The gel was prepared using the ring-cast method at 15 mM concentration and imaged after 48 h. (**c**) Gel–sol transition temperatures of 15 mM halogenated peptide hydrogels formed in 48 h. (**d**) Rheological characterization of the gels by frequency sweep studies whereby the storage modulus (G′) was recorded as a function of angular frequency (*ω*). The peptide DF(I)NKF(I) was determined to be the strongest gel with G′>10^4^ Pa. (**e**) Amplitude sweep studies of peptide hydrogels showing G′ as a function of oscillation strain (*γ*). All halogenated hydrogels show similar linear viscoelastic region (LVR) profiles. Peptide hydrogels at a concentration of 15 mM were utilized for the rheological measurements after 48 h from preparation. For the loss moduli, see the Supplementary Fig. 3.

Hydrogelation is highly indicative of fibrillation, therefore all peptides were in the first instance assessed for their ability to form hydrogels^26^,^27^. All of the studied peptides were found to self-assemble into hydrogels over a minimum gelation concentration. Only the hydrogel of the wild-type peptide DFNKF has been previously reported, though of its *N*-acetylated form at the N terminus^28^. All the reported halogenated peptides are novel types of gelators and, importantly, formed gels (Fig. 2a and Supplementary Table 2) at concentrations much lower than that of their wild-type parent DFNKF, that is, 75 mM, with the super-gelator peptide DF(I)NKF that showed a minimum gelation concentration of <0.2% (2.5 mM; 30-fold lower than DFNKF).

Attempts to study the kinetics of fibril formation using Thioflavin T (ThT) were unsuccessful since no increase in fluorescence was observed upon mixing ThT with peptide solutions. This is well in accordance with the literature since previous studies have demonstrated that DFNKF does not bind this fibril marker dye^29^.

Subsequently, a working concentration of 15 mM was chosen where all halogenated peptides formed clear and elastic gels within a reasonable time (≤12 h). At this concentration, the speed of hydrogel formation followed the order DF(I)NKF(I)>DF(I)NKF≥DF(Br)NKF(Br)>DF(Cl)NKF(Cl)≥DFNKF(I), whereas DFNKF did not form a gel even after 30 days (Supplementary Table 3). The diiodinated peptide formed the most homogeneous and shape-persistent gel (Fig. 2b), whereas peptides DF(Cl)NKF(Cl) and DFNKF(I) showed phase separation of the fibrous aggregates from the aqueous phase. The obtained hydrogels were all thermoreversible with gel–sol transition temperatures ranging from 65 °C (DFNKF(I) to 119 °C (DF(l)NKF(l); Fig. 2c). In particular, the observed transition temperatures followed the same order of the gel forming kinetics reported above. These results clearly show that, in general, halogenation of the DFNKF model peptide greatly promotes hydrogel formation, with the diiodinated peptide being the most efficient. This efficiency decreases with the polarizability of the halogen atom, that is, DF(l)NKF(l)>DF(Br)NKF(Br)>DF(CI)NKF(CI), and also depends on the specific position of the halogen atom in the peptide scaffold, that is, the peptide iodinated at the Phe in the second position is more efficient than the one iodinated in the fifth.

Characterization of the different halogenated peptide hydrogels was done by oscillatory rheology (ring-cast method) using the 15 mM concentration. At this low concentration, the wild-type peptide DFNKF only formed a viscous fluid and was not studied further. The diiodinated peptide DF(I)NKF(I) was confirmed to form the stiffest gel, which is reflected in its high elastic modulus (G′>10^4^ Pa and G′>>G′′; Fig. 2d and Supplementary Fig. 3) and by the fact that the gel assumes the well-defined shape of the mould (Fig. 2b). The G′ value for DF(I)NKF(I) is comparable to that of Ac-LIVAGD, a hexamer that forms one of the best performing hydrogels in terms of ease of hydrogel formation and strength^30^,^31^. Interestingly, the trend of G′ values parallels those of gel formation efficiency and thermal stability whereby gels of peptides DF(I)NKF(I), DF(I)NKF and DF(Br)NKF(Br) are the stiffest and DFNKF(I) and DF(Cl)NKF(Cl) formed the weakest ones.

**Figure 3:**
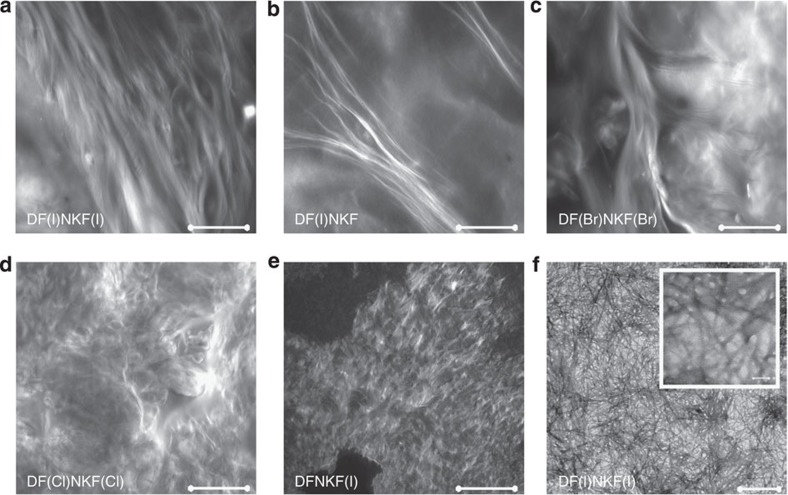
Fibrillar morphologies of halogenated hydrogels. (**a**–**e**) Confocal microscopy images of peptide hydrogels at a concentration of 15 mM upon aging 48 h at RT and after staining with Rhodamine B (scale bar, 100 μm). Panels **a**, **b** and **c** show bundles of long twisted fibrils belonging to hydrogels of peptides DF(I)NKF(I), DF(I)NKF and DF(Br)NKF(Br), respectively. Panels **d** and **e** show much smaller fibrils of hydrogels of peptides DF(Cl)NKF(Cl) and DFNKF(I), which intertwine and form a matrix-type structure. (**f**) TEM image of the dried hydrogel of peptide DF(I)NKF(I) showing an intertwined network of long fibrils (scale bars, 0.5 μm and 5 nm).

Morphology and microarchitecture of the self-assembled fibrillar networks constituting the halogenated hydrogels were evaluated by confocal microscopy (Fig. 3)^32^. The network of the hydrogel of DF(I)NKF(I) appeared to be very filamentous with micrometre-long fibrils assembled in bundles with a helical sense, which propagate along the same direction. These micrometre-long fibrils become fewer and thinner on going from DF(I)NKF(I) to DF(I)NKF and DF(Br)NKF(Br), and disappear in the hydrogels of DF(CI)NKF(CI) and DFNKF(I) where only entangled matrices of small fibrils and aggregates were observed. These observations fit well with the rheology data where the strongest gels result from the entanglement of the longest fibrils. In agreement with the confocal microscopy study, the hydrogel formed by the peptide DF(I)NKF(I) showed an intertwined network of long fibrils in its transmission electron microscopy (TEM) image (Fig. 3f).

**Figure 4:**
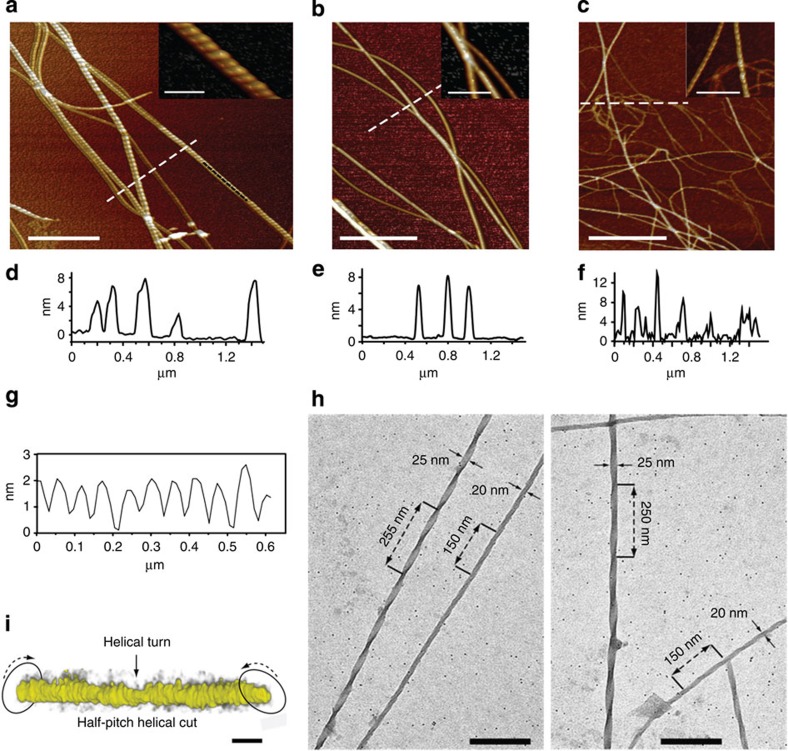
Nanofibrillation of the halogenated pentapeptides. AFM image close-up insets of (**a**) peptide DF(I)NKF(I), (**b**) peptide DF(I)NKF, and (**c**) peptide DF(Br)NKF(Br) evaporated on mica substrates after 9 days incubation in aqueous solutions (scale bar, 1 μm). (**d**–**f**) Height profiles of fibrils from **a**, **b** and **c** (cross-section lines highlighted in white). (**g**) Cross-sectional analysis on the top of a fibril segment in **a** showing a helicoidal profile along the longitudinal direction of the fibrils (highlighted in black). (**h**) Cryo-TEM images of the dried hydrogel of DF(I)NKF, showing a network of long fibrils (scale bar, 200 nm). (**i**) *In situ* electron tomography of half-pitch helicoidal peptide DF(I)NKF(I) vitrified from aqueous solution. The twisted fibril morphology is highlighted by arrows. Electron tomography reconstructions were collected upon tilting of samples up to ±69° (scale bar, 25 nm).

The nanostructure of the halogenated fibrils was revealed by atomic force microscopy (AFM) (Fig. 4). The peptides were first imaged after incubation in dilute aqueous conditions for 9 days (see also Supplementary Fig. 4) and Fig. 4a–c highlight the most strongly fibrillating peptides DF(I)NKF(I), DF(I)NKF and DF(Br)NKF(Br). While only small spherical aggregates were observed for the wild-type peptide DFNKF (Supplementary Fig. 4f), all of the halogenated peptides showed pronounced fibrillar structures. Large differences were, however, observed between the monoiodinated peptides DFNKF(I) and DF(I)NKF: Peptide DFNKF(I) (Supplementary Fig. 4e) showed only small protofibrils and aggregates, whereas the peptide DF(I)NKF (Fig. 4b and Supplementary Fig. 4c) showed large twisted fibrils similar to those of the diiodinated peptide DF(I)NKF(I) (Fig. 4a and Supplementary Fig. 4a), which, in turn, had the most complex structures with twisted ribbon-like fibrils tens of nanometre wide and micrometre long. These results highlight the importance of the specific position of the iodine atom in the peptide structure and therefore the subsequent specific interactions, which result in different amyloid fibril topographies. That the fibrillation and twisting is not due to drying is excluded by high-resolution cryo-TEM and electron tomography (ET) studies. This is exemplified for the peptide DF(I)NKF(I) corresponding to the strongest hydrogel. It shows fibrils with lateral sizes of 20–25 nm and helical twisting with periodicities between 250 and 140 nm (Fig. 4h and Supplementary Fig. 4b). Such periodicities correspond to the different maturation steps of amyloid fibrils as previously reported for β-lactoglobulin fibrils^33^. ET allowed three-dimensional visualization of the twisting morphology of a DF(I)NKF(I) fibril imaged in solution (Fig. 4i).

### Amyloid structure of fibrils of halogenated peptides

There are three criteria that define a protein aggregate as an amyloid fibril: green birefringence upon staining with Congo Red, fibrillar morphology and β-sheet secondary structure^34^. Therefore, Congo red staining was carried out for all the peptides (at the same 15 mM concentration) and strong green-gold birefringence was observed under polarized light, which is a reporter for long-range cross-β structure (Supplementary Fig. 5).

Further evidence of amyloid nature was obtained by Fourier transform infrared (FT-IR) spectroscopy. The FT-IR spectra of both the solution and the gel state in D_2_O were recorded (Supplementary Fig. 6). A solution of the wild-type peptide DFNKF showed no significant amide I’ band, however, using a concentration at which it forms a gel (75 mM), this band became visible. This peak at around 1,630 cm^−1^ can be attributed to the peptide bond carbonyl upon formation of the amyloid β-sheet structure and subsequent gelation^35^. The spectra of the gel state of the halogenated peptides showed this same amide I’ band consistent with the formation of amyloid fibrillar species comprising β-sheet elements. These data confirm that all the studied halogenated derivatives of DFNKF form fibrils of the same amyloid nature to their wild-type parent. Interestingly, this 1,630 cm^−1^ peak is red-shifted in the spectra of DF(I)NKF(I) and DF(I)NKF, while it is not in the one of DFNKF(I) (Supplementary Fig. 6). This red-shift indicates a reduced electron density on the peptide bond and is consistent with the involvement of the carbonyl oxygen in halogen bonding^36^.

Raman spectroscopy is a powerful tool to investigate the occurrence of the halogen bond^37^. For this reason, we followed the changes experienced by the C–I stretching band at around 167 cm^−1^ upon formation of the gel. Interestingly, compared with the bulk powders of the starting materials, in the dried gels of the iodinated peptides DF(I)NKF(I) and DF(I)NKF, this band shifted to lower frequency (see Supplementary Fig. 7). This shift is perfectly consistent with the weakening of the C–I bond as a consequence of its involvement in halogen bonding upon formation of the hydrogels^37^.

### Co-assembly of wild-type peptide with diiodinated analogue

**Figure 5:**
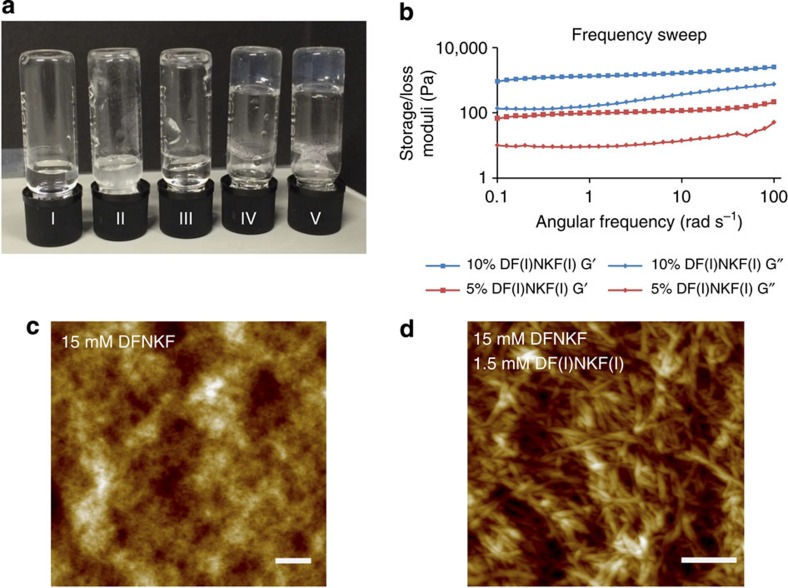
Mixed hydrogels formed by co-assembly of DFNKF and DF(I)NKF(I). (**a**) Photograph of vials after 48 h containing (I) 15 mM DFNKF, which did not form hydrogel; (II) 1.5 mM DF(I)NKF(I), which did not form hydrogel and some precipitation was observed; (III) 15 mM DFNKF:0.15 mM DF(I)NKF(I), which did not form hydrogel; (IV) 15 mM DFNKF:0.75 mM DF(I)NKF(I), which formed hydrogel within 48 h; (V) 15 mM DFNKF:1.5 mM DF(I)NKF(I), which formed hydrogel within 48 h. (**b**) Rheological characterization of mixed hydrogels by frequency sweep studies whereby the storage concentration of 15 mM after 48 h were utilized for the rheological measurements. (**c**) AFM of a 15 mM solution of wild-type peptide DFNKF showed small fibrils and aggregates unable to network sufficiently to form a hydrogel. (**d**) AFM of a sample of the mixed hydrogel containing 15 mM DFNKF and 1.5 mM DF(I)NKF(I) showed larger and more entangled fibrils (scale bar, 400 nm).

Bromination and chlorination of proteins *in vivo* has been related to a series of oxidative stress-related diseases such as cystic fibrosis^38^, atherosclerotic intima^39^, sepsis^40^ and asthma, among others^41^. A direct correlation exists between the role of oxidative stress and protein fibrillation^42^. However, how halogenation affects protein structure, folding and functioning is not yet known. To study to what extent a partial degree of halogenation may promote the amplified formation of amyloid fibrils, we studied the co-assembly^43^ of the strongly self-assembling diiodinated peptide DF(I)NKF(I) with the wild-type peptide DFNKF. Mixing DFNKF and DF(I)NKF(I) at concentrations at which normally neither form gels alone (Fig. 5a), 15 and 0.75 mM (5%), respectively, resulted in a mixed hydrogel within 18 h.

Characterization of the physical properties of the mixed hydrogel was performed by oscillatory rheology (ring-cast method) where the mixed hydrogel was found to be weaker than the hydrogel containing only DF(I)NKF(I) (Fig. 5b versus Fig. 2d). It was also observed that increasing the amount of the strong gelator DF(I)NKF(I) led to an increase in the elastic modulus value, whereas the gel containing 10% was stronger than the one containing 5% of DF(I)NKF(I). Interestingly, circular dichroism (CD) analysis of the mixed hydrogels showed the same structural features observed in the CD spectra of the pure iodinated peptides (see Supplementary Figs 8 and 9).

The resulting fibril morphologies in the mixed hydrogels were studied by AFM (Fig. 5c,d). A 15 mM sample of the wild-type peptide DFNKF showed only a mixture of intertwined aggregates and small fibrils, which is consistent with the observation that no gelation is observed at this concentration since the fibrils appear too small to crosslink and form a network (Fig. 5c). On the addition of 10% DF(I)NKF(I), the resulting fibrils were much longer and intertwined to form a fibrous network resulting in the hydrogelation of the wild-type peptide DFNKF solution (Fig. 5d and Supplementary Fig. 10a). The weakness of the resulting mixed hydrogel compared with that of DF(I)NKF(I) alone can be explained by the relative size and network formation of the fibrils (Supplementary Fig. 10b). This result may have important implications in a biological setting since only small amounts of brominated or chlorinated peptides may be required to amplify the supramolecular self-assembly of an amyloid fibril.

### Role of halogen bonding in hydrogels’ self-assembly

**Figure 6:**
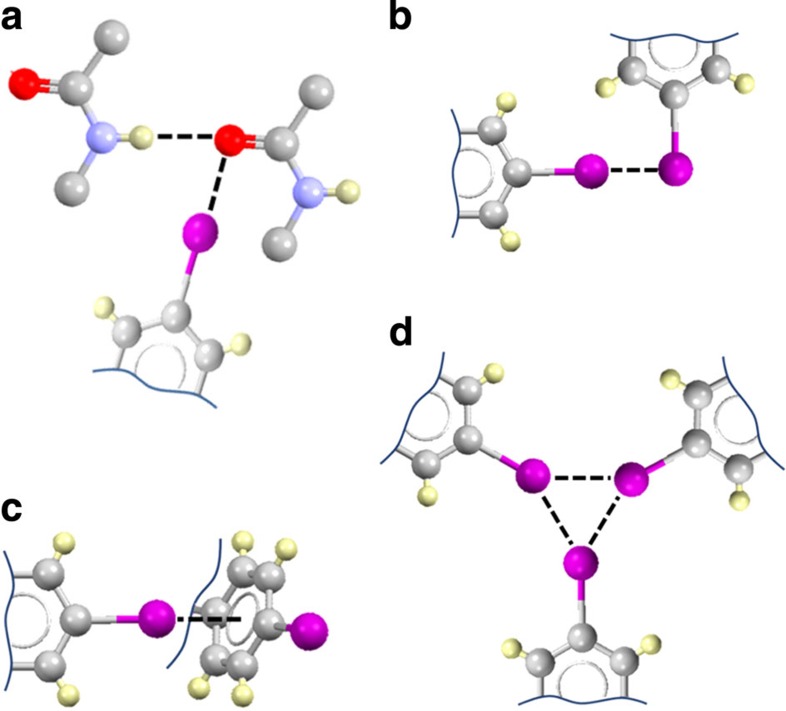
Potential halogen bonds involving *p*-iodo-phenylalanine. IR and Raman spectroscopies are more consistent with the occurrence of type **a** interaction, however, other interactions reported cannot be discounted. (**a**) Orthogonal hydrogen and halogen bonds involving the carbonyl oxygen atom of the peptide bond^36^. (**b**) Type II iodine···iodine contacts^23^. (**c**) Iodine···π interactions^49^. (**d**) Triangular iodine synthon^50^.

Although no direct structural evidence of halogen bonding between our peptide monomers has been obtained, yet, the reported results, IR and Raman in particular, provided corroborative evidence that iodinated peptides DF(I)NKF and DF(I)NKF(I) potentially use halogen bonds to efficiently self-assemble into mature amyloid fibrils. In fact, the particular position of the iodinated residue in the peptide sequence greatly influences the efficiency of its self-assembly process, thus suggesting the involvement of the iodine atom in a specific noncovalent interaction (Fig. 6a), rather than nonspecific hydrophobic and/or steric effects. This is further supported by considerations of halogen atom polarizability since the dibrominated and dichlorinated derivatives DF(Br)NKF(Br) and DF(Cl)NKF(Cl) showed less tendency to form fibrils than their diiodinated analogue DF(I)NKF(I). This result allows interactions involving halogen atoms as hydrogen bond acceptors to be ruled out as Cl and Br would give stronger interactions than I, which was not observed. Electrostatic interactions involving the ionizable side chains of the peptides, Asp and Lys, may also be ruled out as the formation of these halogenated hydrogels is largely pH-independent (see Supplementary Table 4). As a confirmation of the minor role played by electrostatic attraction in the self-assembly of the reported halogenated peptides, increasing the ionic strength of the peptide solutions resulted in accelerated formation of the hydrogels (see Supplementary Table 5). It is also unlikely that aromatic π–π interactions are playing an important role in the self-assembly of the halogenated hydrogels, since this would not explain the polarizability dependence that we observed. Finally, hydrophobic interactions could not justify the observed trends in hydrogel properties because DF(Br)NKF(Br) and DF(Cl)NKF(Cl) are both more hydrophobic than DF(I)NKF (see Supplementary Table 6), which instead forms the second stiffest gel. Moreover, the two monoiodinated peptides, despite having similar hydrophobicity, display completely different properties. For the same reasons discussed above, electrostatic and hydrophobic interactions also should not play an important role in the interfibrillar association observed in the halogenated gels.

## Discussion

In summary, we have demonstrated that iodination of a short amyloidogenic core sequence strongly promotes its fibril formation ability and affects the structure of formed fibrils. In particular, the 2-*p*-iodo-Phe derivative DF(I)NKF was demonstrated to be 30-fold more efficient in forming hydrogels than the wild-type pentapeptide DFNKF. This is remarkable for such a modest perturbation in structure. Preliminary results indicate that the strong effect of iodination on peptide self-assembly is general and not limited to the core sequence reported here. In fact, iodination of the amyloidogenic core sequence KLVFF (residues 16–20 of Aβ) as well as of the full-length hCT similarly promotes fibril formation efficiency and self-assembly (data not shown). Iodination of aromatic amino acids may thus develop as a general strategy for the design of new hydrogels starting from unprotected peptides and without the use of organic solvents^44^,^45^.

The results reported in this paper are relevant in the field of the programmed synthesis of amyloid supramolecular assemblies as well as in the context of amyloid-dependent diseases. In the former field, we have demonstrated that single atom mutations of amyloidogenic core sequences by replacing hydrogen with iodine atoms greatly promote fibril formation and self-assembly. Methods promoting the formation of mature fibrils are advantageous for use, among others, as scaffolds for tissue engineering, controlled drug release, surgical reconstruction applications, microfluidic devices, biosensors and bioswitches^7^,^8^,^9^,^10^,^11^. As far as amyloid-dependent diseases are concerned, our results suggest that oxidative stress-induced halogenation of proteins might potentially be the triggering point for the transformation of a natively folded protein into a halogen-bonded and fibrillar malfunctioning form. Research in this direction is currently being carried out in our laboratories and will be reported elsewhere.

## Methods

### Reagents

Congo red, Rhodamine B, NaCl and D_2_O were purchased from Sigma-Aldrich and used without further purification. Peptides with confirmed amino acid analysis (purity ≥98%), were purchased from Biopeptek (Malvern, USA). The integrity of all peptides was confirmed by ion spray mass spectrometry and the purity was determined by reverse phase high-pressure liquid chromatography.

### Hydrogel preparation

Hydrogels were prepared by dissolving each peptide (15 mM for halogenated peptides and 75 mM for the wild-type peptide DFNKF) in deionized water (18.2 Mω cm) or in D_2_O (≥99.9 atom % deuterium). The glass vials containing the 500 μl solutions were sealed, sonicated for 20 s, heated using a heat gun until complete dissolution of the peptides, before slow cooling to room temperature (RT) All the samples were stored at RT for 48 h before analysis.

### Preparation of peptide solutions

Peptide solutions (40 μM) were freshly prepared in deionized water (18.2 Mω cm), sonicated for 20 s, and gently warmed to reach 90 °C before filtration through a 0.22 μm Millipore filter. The peptide solutions were stored in sealed vials at RT for varying time points before analysis.

### X-ray crystallography

The *p*-iodo-phenylalanine was suspended in water at RT and kept in an open vial under a hood. After 6 months, colourless crystalline needles appeared. Suitable crystals for XRD measurements were analysed without further manipulation. The crystals were measured using Mo-Kα radiation on a Bruker KAPPA APEX II diffractometer with a Bruker KRYOFLEX low temperature device. The crystal structure was solved by direct method and refined against F^2^ using SHELXL97^46^. Packing diagrams were generated using Mercury^47^. The non-hydrogen atoms were refined anisotropically and hydrogen atoms were fixed geometrically and refined isotropically (CCDC deposition number: 1049844).

### Mixed gels preparation

Mixed hydrogels were prepared using the same method as previously described. Briefly, peptides were weighed into a glass vial and MilliQ water added. The glass vial was sealed and sonicated for 20 s before heating with a heat gun until complete solubilization of the peptides. The vials were allowed then to cool slowly to RT and stored at RT for 7 days before analysis.

### Thermal stability

The vials containing the hydrogels at 15 mM were inverted and fixed at the bottom of an oil bath with stirring. The temperature was kept at 25 °C for 10 min to equilibrate the system and then gradually increased from 25 to 140 °C (at 1 °C min^−1^). The temperature at which the gels break is reported as a range: the initial temperature corresponds to the fall of the first drop and the final one to the complete breakdown of the gel.

### Rheology

Rheology experiments were performed using a TA instrument ARG2 Rheometer. A 20 mm stainless steel, parallel-plate geometry was used with a gap distance of 1,000 μm. Oscillatory frequency sweep studies were performed for a range of 0.1–100 rad s^−1^, using a 0.5% strain. Oscillatory amplitude sweep studies were conducted from 0.01 to 100% strain with an angular frequency of 1 rad s^−1^. The ring-cast method was used for hydrogel preparation at a peptide concentration of 15 mM. The peptide solution was sonicated for 20 s in a sealed glass vial before heating to 90 °C to afford complete dissolution of the peptide. After cooling, the solutions were transferred into ring casts of 22 mm diameter and placed in tightly sealed tissue-culture dishes for 48 h. All the measurements were repeated a minimum of three times.

### Circular dichroism spectroscopy

All the CD experiments were carried out in deionized water (18.2 MΩ cm) in a 0.01 mm detachable quartz cuvette, using a JASCO J-815 CD spectrometer. Acquisitions were performed between 190 and 300 nm with a 0.1 nm data pitch, 1 nm bandwidth, 100 nm min^−1^ scanning speed and 1 s response time. All the spectra are an average of 10 scans and were corrected from a reference solution, comprising deionized water (18.2 MΩ.cm) alone. Raw data (*θ*, in millidegree) were subsequently converted to mean residue ellipticity ([*θ*] in deg cm^2^ dmol^−1^) for the sake of comparison, in accordance with the following formulae:




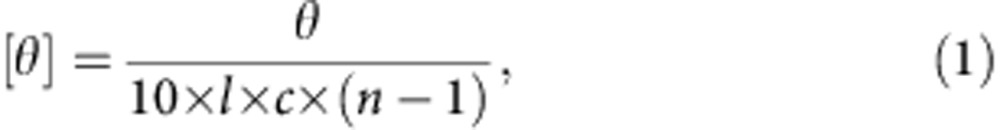




where *θ* is the observed ellipticity in millidegree, *c* is the concentration of the sample in mol l^−1^, (*n*−1) is the number of peptide bonds and *l* is the path length of the cuvette in centimetre.

### Confocal microscopy

Hydrogels were imaged using a Zeiss LSM 710 microscope with a He/Ne laser (*λ*_ex_=543 nm). The fluorescent dye, Rhodamine B, was incorporated into an aged hydrogel (48 h) scaffold by the addition of 10 μl of the dye solution (0.1% w/v). Following complete absorption of the dye, the sample was excited at 543 nm and emitted light recorded using the E570LP emission filter.

### Congo red staining

All the samples were monitored for green birefringence using an Olympus BX50 polarizing microscope with a SensiCam PCO camera used to display and enhance images. An 80% ethanol solution saturated with NaCl and Congo red was freshly prepared before each measurement. A piece of each peptide hydrogel was placed on a glass microscope, allowed to air dry and then stained with Congo red solution. Subsequently, excess Congo red solution was blotted off the slide and the samples were analysed using both bright and polarized light.

### Infrared spectroscopy (FT-IR)

Infrared spectra were recorded at RT using a Nicolet iS50 FT-IR spectrometer equipped with a DTGS detector. Peptides were analysed as solutions (after heating at 100 °C to break any pre-formed fibrils) or gels at 15 mM in D_2_O (for DFNKF 75 mM concentration was used). Spectra represent an average of 64 scans recorded in a single-beam mode with a 4 cm^−1^ resolution and corrected for the background. The second-derivative analyses of the spectra were performed using the Nicolet FTIR software, Omnic 9.0, with a 13-point and third polynomial order Savitzky and Golay function. Second-derivative spectra generated negative bands as compared with the original spectra, thus for comparison all the second-derivative spectra were multiplied by −1.

### Raman spectroscopy

Raman spectra were acquired at RT by using a Horiba Xplora MicroRaman instrument equipped with an Olympus BX-41 Microscope. An excitation wavelength of 785 nm was used. Laser power was attenuated by neutral density filters with a final power density of ∼0.07 mW μm^−2^. The low wavenumber detection limit is 140 cm^−1^. Each spectrum was acquired with an exposure time of 5 s over 35 cycles. The raw data were first corrected from the baseline, using the JASCO Nicolet FTIR software, Omnic 9.0, between 140 and 340 cm^−1^. Obtained data were subsequently normalized, for the sake of comparison, and plotted using Origin Pro 8.

### Atomic force microscopy

Peptide solution (10 μl, 40 μM) or diluted gel was deposited onto freshly cleaved mica surface and air dried. AFM characterizations were performed on a Veeco Dimension 5,000 Scanning Probe Microscope with a Nanoscope V controller (Digital Instruments, Inc.). All the samples were prepared on mica substrate and measured without treatment. Al-coated silicon AFM tips (NSC 15/AIBS, MikroMasch, Estonia) with a tip radius of 10 nm were used to probe the surface profiles of the films. Tapping-mode AFM imaging was used according to well-established procedures. All the images were post-treated with NanoScope Analysis 1.5 Software.

### Transmission electron microscopy

High-resolution transmission electron microscopy imaging was carried out using JEM-3200Fsc field emission microscope (JEOL) operated at 300 kV in bright-field mode with Omega-type Zero-loss energy filter. The images were acquired with ULTRASCAN 4,000 CCD camera (GATAN) and with GATAN DIGITAL MICROGRAPH software, while the specimen temperature was maintained at −187 °C. Dried TEM and ET samples were prepared by placing 4 μl of 40 μM DF(I)NKF(I) solution on 200 mesh carbon only grid (CFT200-Cu) and the excess removed with filter paper.

### Electron tomography

For image alignment purposes, the TEM grids were dipped in 11-mercapto-1-undecanol ligand-coated gold particle solution (*d*=3–10 nm) before sample deposition. ET tilt series were acquired with the SERIALEM-software package between tilt angles of ±69°. Prealignment of tilt image series was done with IMOD and the fine alignment and cropping with JPEGANIM software package^48^. The images were binned twice to reduce noise and computation time. Maximum entropy method reconstruction scheme was carried out with MEM software package^48^ on Linux cluster with regularization parameter value of *δ*=5.0 × 10^−2^. Data visualization, volumetric graphics and analyses were performed with the UCSF CHIMERA package. The tomogram was filtered with CHIMERA’s Gaussian filter with 1.5 voxel width.

## Additional information

**Accession codes:** The X-ray crystallographic coordinates for the structure reported in this study have been deposited at the Cambridge Crystallographic Data Centre (CCDC), under deposition number 1049844. These data can be obtained free of charge from The Cambridge Crystallographic Data Centre via www.ccdc.cam.ac.uk/data_request/cif.

**How to cite this article:** Bertolani, A. *et al.* Supramolecular amplification of amyloid self-assembly by iodination. *Nat. Commun.* 6:7574 doi: 10.1038/ncomms8574 (2015).

## Supplementary Material

Supplementary Figures, Supplementary Tables and Supplementary References Supplementary Figures 1-10, Supplementary Tables 1-6 and Supplementary References

Supplementary Data 1 Crystallographic information file for p-iodo-phenylalanine

Supplementary Data 2 Check CIF report for p-iodo-phenylalanine CIF
