# Supplementary material for: Supramolecular amplification of amyloid self-assembly by iodination
Source: Nat Commun. 2015 Jun 30;6:7574. doi: 10.1038/ncomms8574 (PMC4491812; doi:10.1038/ncomms8574)
Supplement: Supplementary Figures, Supplementary Tables and Supplementary References — Supplementary Figures 1-10, Supplementary Tables 1-6 and Supplementary References [file ncomms8574-s1.pdf]

Supplementary Figures

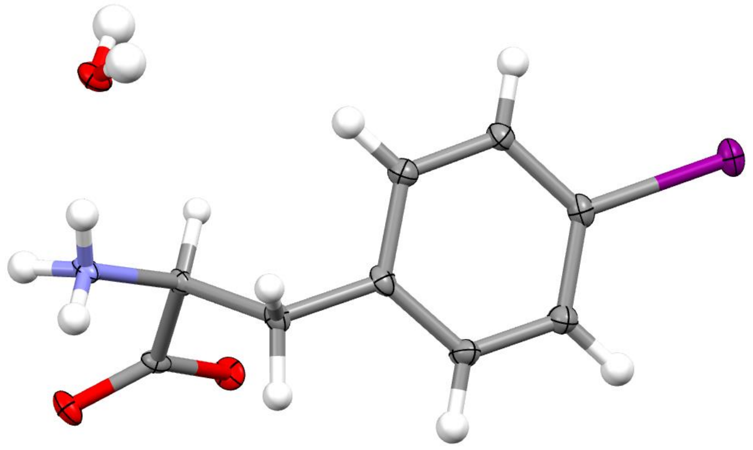

Supplementary Figure 1. The asymmetric unit in *para*-Iodio-Phenylalanine crystal.† The 50% probability ellipsoid representation was prepared using the Mercury Software. Colors are as follows: C, grey; H: white; O: red; N: blue; I: magenta.

† See Supplementary data 1 for the CIF file.

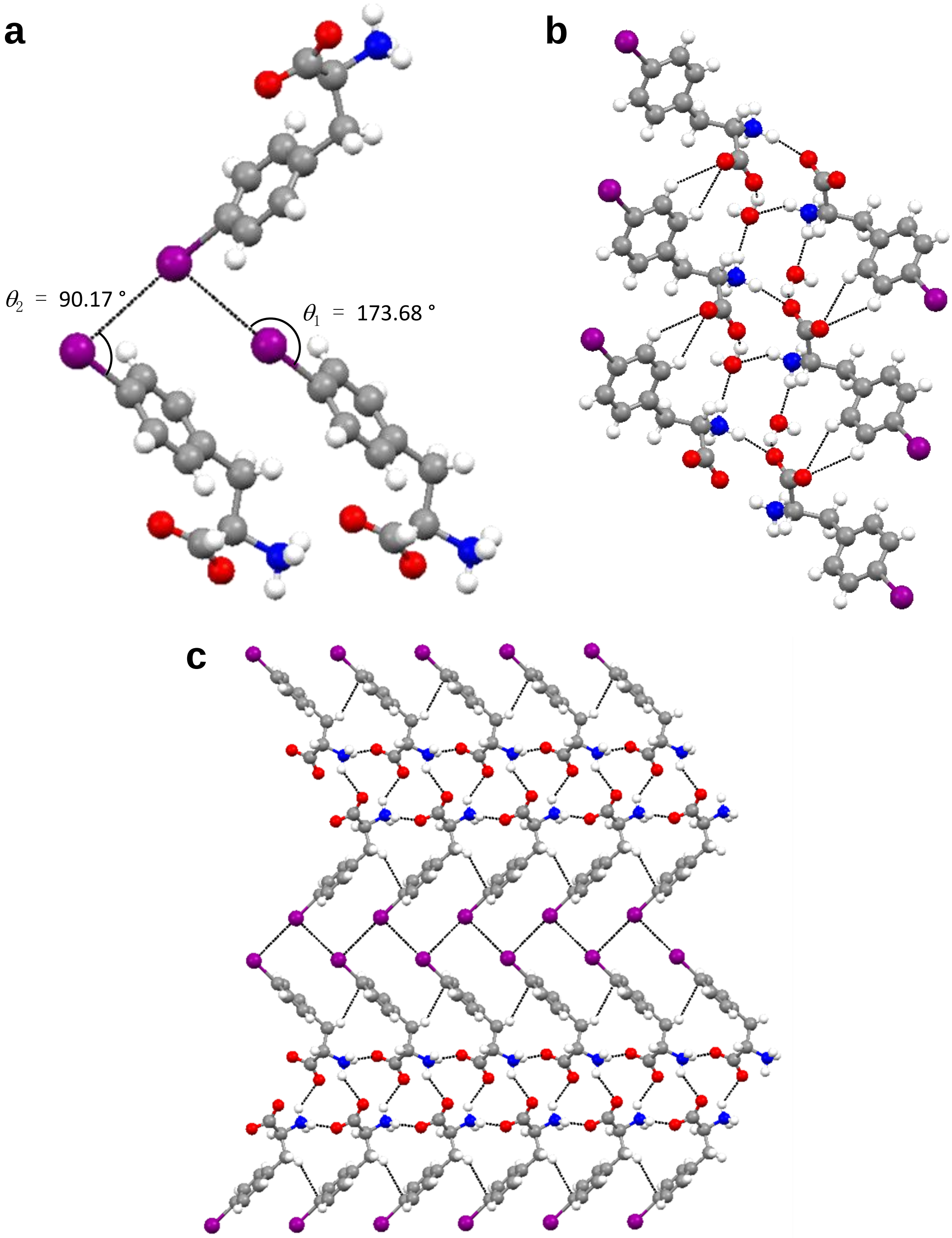

Supplementary Figure 2. Single crystal X-ray structure of *p*-iodo-phenylalanine. a, The lateral assembly of *p*-iodo-phenylalanine showing type II iodine···iodine contacts ($\theta_1 \cong 180°$, $\theta_2 \cong 90$ °). **b**, Hydrogen bonding network of the amino acid showing intermolecular interactions between two strands and involving the amino and carboxyl termini. **c,** Crystal packing of *p*-iodo-phenylalanine. Colours are as follows: C, grey; H: white; O: red; N: blue; I: magenta; hydrogen and halogen bonds are dashed black lines.

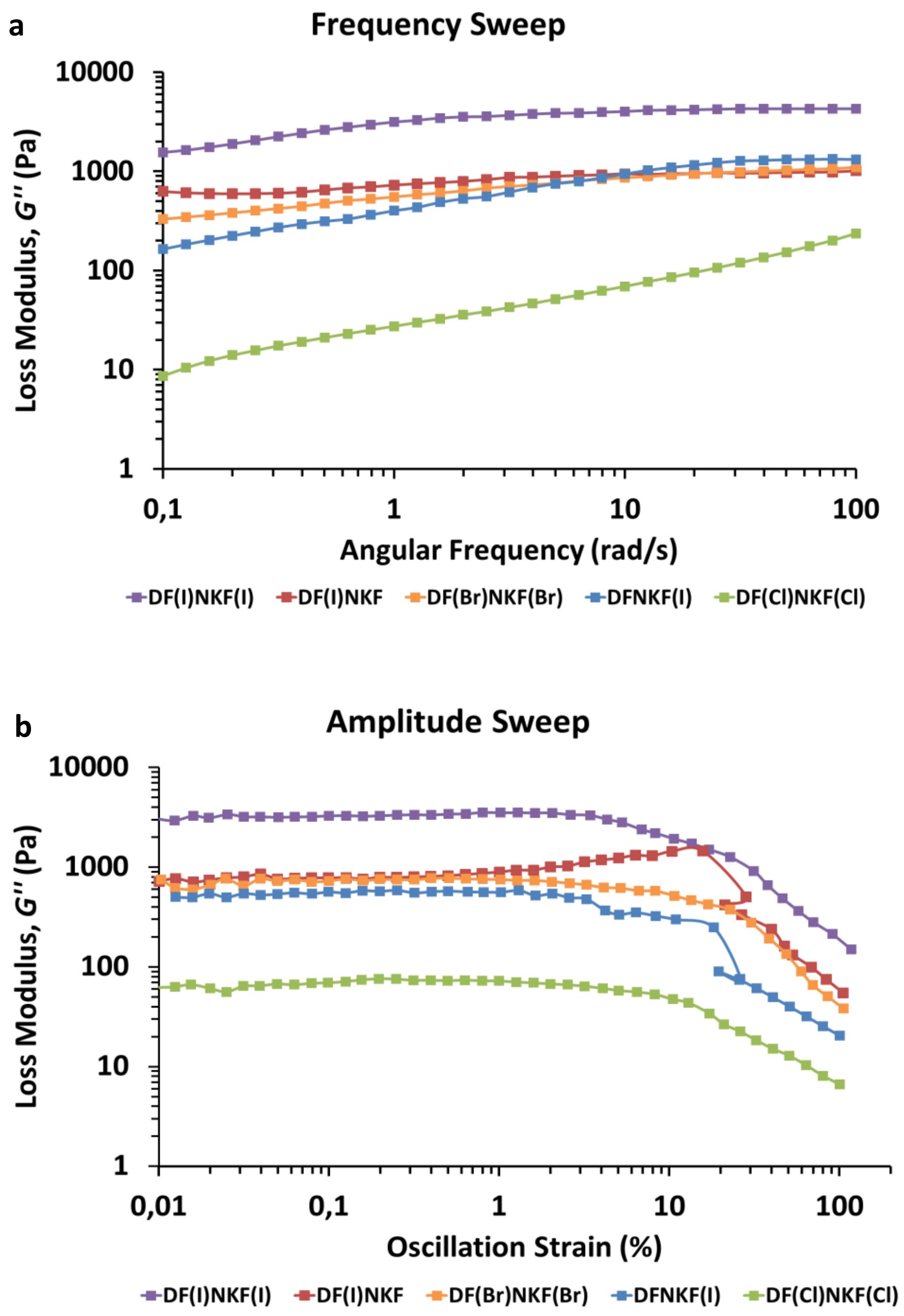

Supplementary Figure 3. Characterization of halogenated hydrogels at 15 mM by oscillatory rheology (ring-cast method). a, Frequency sweep showing the variation of loss modulus (G'') as a function of angular frequency (ω). The trend is the same as observed for the storage modulus (G') (see main text). Amplitude sweep measuring the variation of G'' with increasing oscillation strain (γ). The trend is the same as observed for G' and also follows the same order as the frequency sweep studies. Peptide hydrogels at a concentration of 15 mM were utilized for the rheological measurements after 48 hours from their preparation.

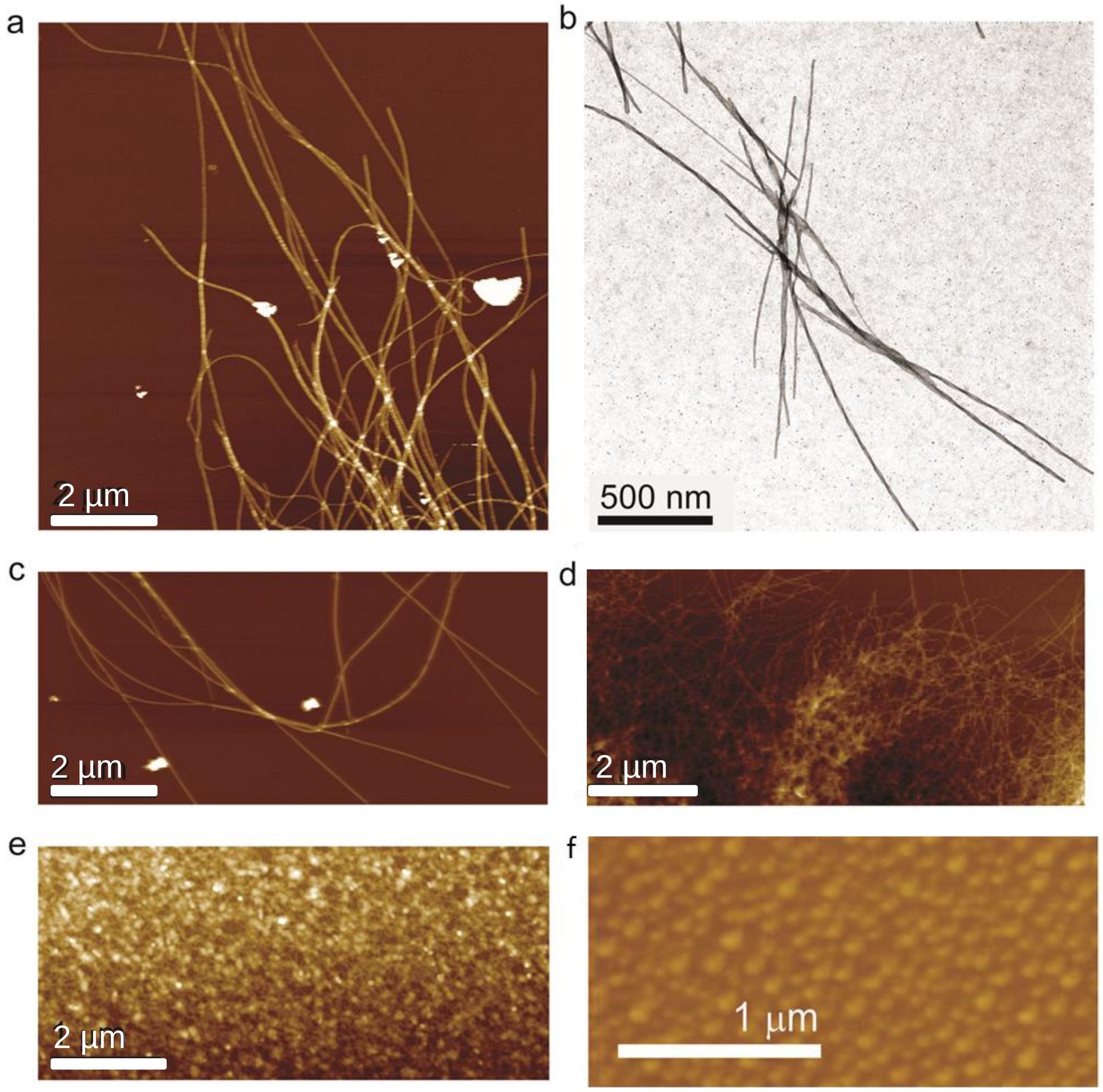

Supplementary Figure 4. Microscopy of amyloid fibrils. Large-scale AFM images of **a.** DF(I)NKF(I), **c.** DF(I)NKF, **d.** DF(Br)NKF(Br), **e.** DFNKF(I), and **f.** DFNKF evaporated on mica substrates from aqueous solutions after a 9 days incubation period at r.t. **b.** An additional TEM micrograph of DF(I)NKF(I) showing twisted fibrils with different size and helical pitch length.

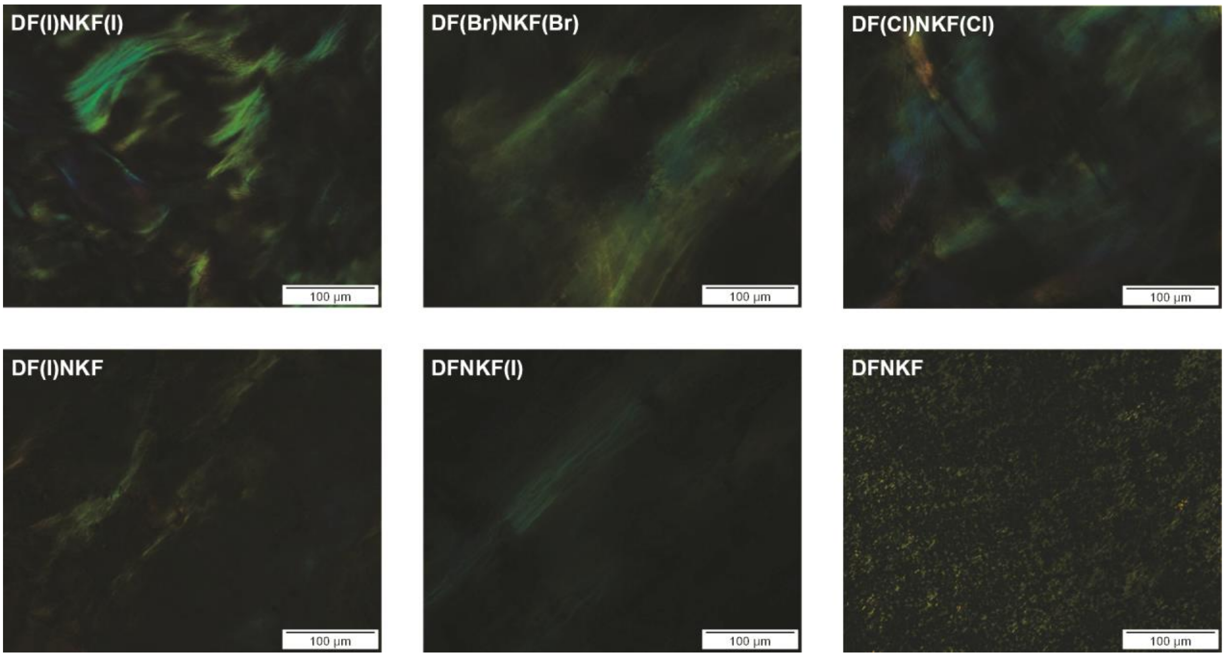

Supplementary Figure 5. Congo red staining of peptide fibrils. Optical microscopy images of all of the studied peptides upon staining with Congo red and visualized under polarized light. In all cases, the clear green birefringence confirms the formation of amyloid fibrils.

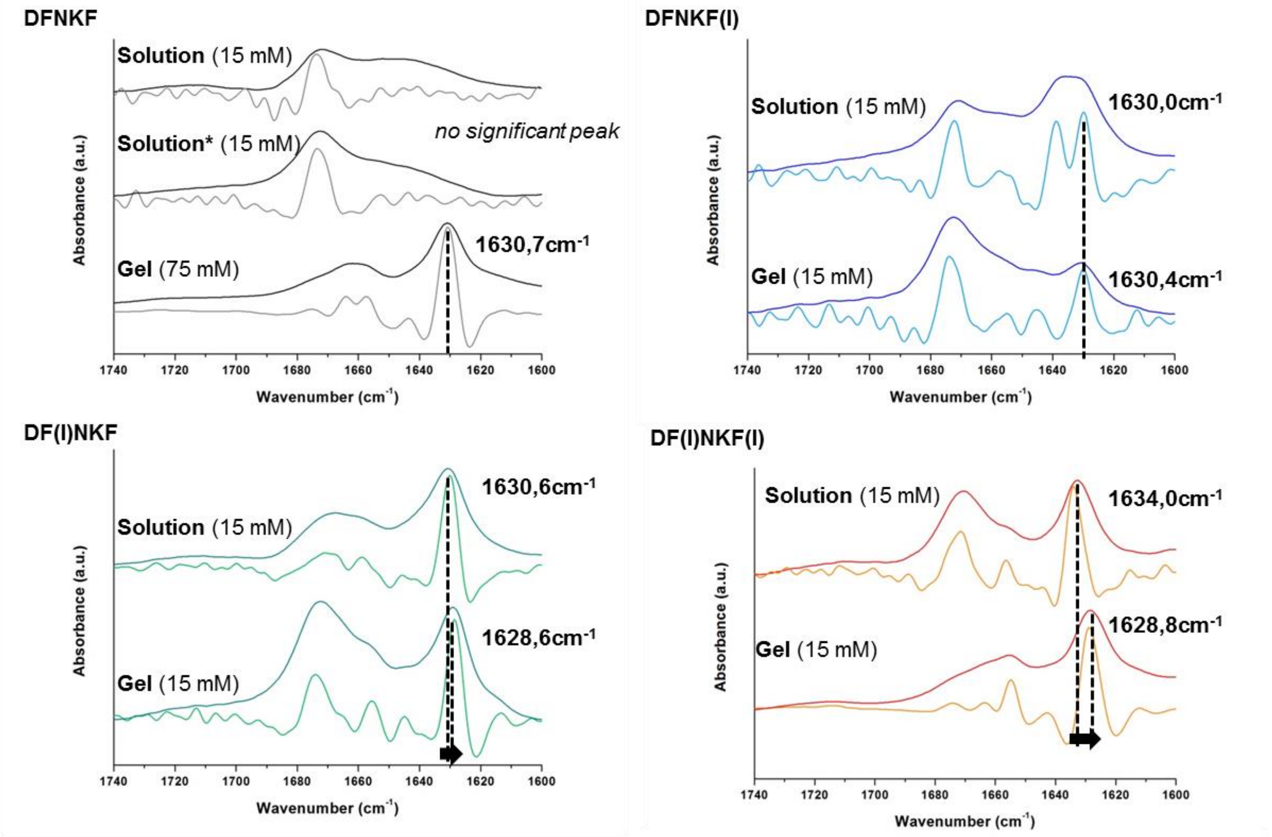

Supplementary Figure 6. UATR FT-IR spectroscopy of peptide solutions and hydrogels. IR spectra of peptides DFNKF, DFNKF(I), DF(I)NKF, and DF(I)NKF(I). Solutions at 15 mM were used for the solution spectra and the gel spectra were recorded using the same samples after standing for 48 hours at r.t. At this concentration the wild-type peptide DFNKF did not form a gel, even after heating and standing for 48 hours at r.t. (solution* spectrum). The lack of the amide I’ band in the solution state could be due to the low sensitivity of the IR technique. However, using a concentration at which the wild-type peptide formed a gel (75 mM), this band became visible. Peptides DF(I)NKF and DF(I)NKF(I) clearly showed a redshift of the amide I’ band upon gelation (fibril formation). This redshift was not observed in the case of the peptide DFNKF(I). The spectra are reported as raw spectra (top spectrum) and as the corresponding 2nd derivatives (bottom spectra).

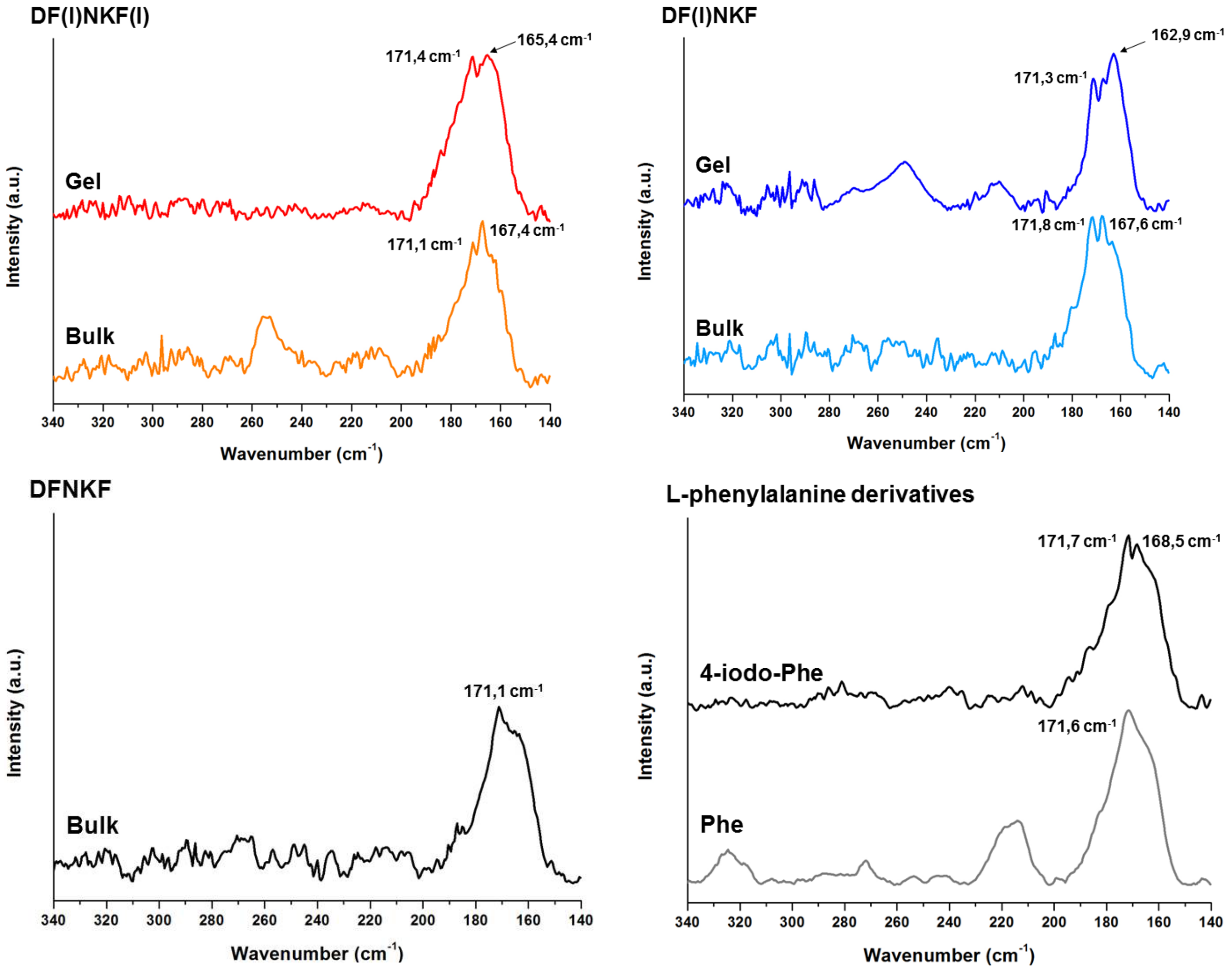

Supplementary Figure 7. Raman spectroscopy of peptide bulk powders and dried hydrogels. Raman spectra of peptides DF(I)NKF(I), DF(I)NKF, and DFNKF, together with L-phenylalanine derivatives (Phe and *p*-I-Phe). Peptides DF(I)NKF(I) and DF(I)NKF were analysed as dried gels (from solution of 15 mM used after standing 48h at r.t.) and as bulk powders. The wild-type peptide DFNKF and the phenylalanine derivatives were analysed directly as bulk powders.

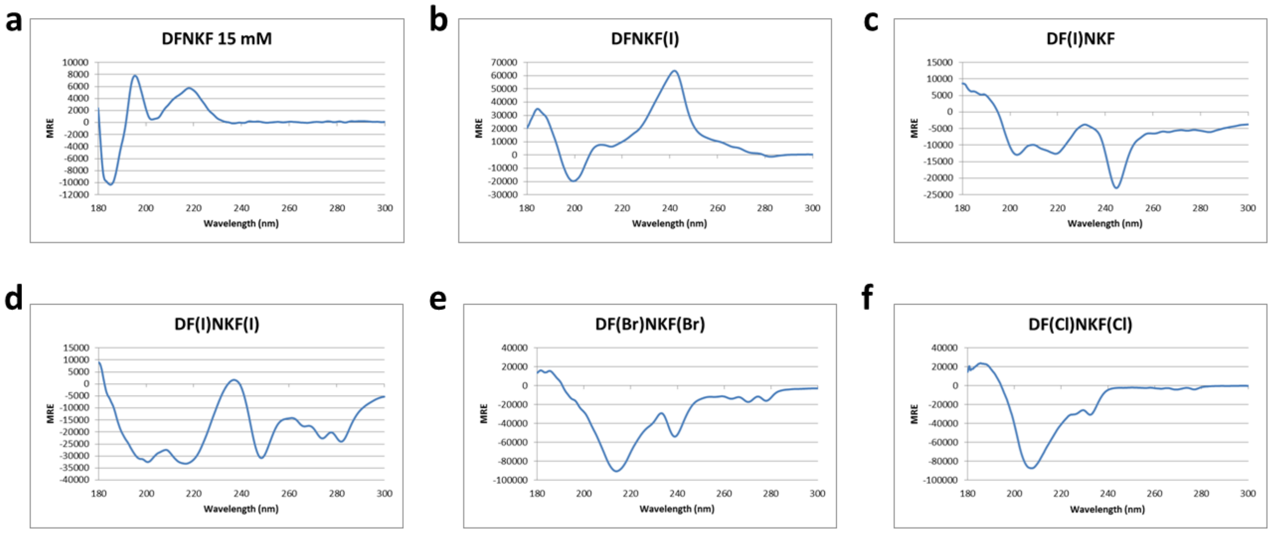

Supplementary Figure 8. Circular Dichroism spectra of peptide hydrogels (solution for DFNKF). All the spectra were recorded at 15 mM concentrations. It is well known that the presence of aromatic groups in a peptide sequence can perturb CD signals related to secondary structure since n-π* and π-π* transitions between aromatic groups also absorb in the same region.[1] It can, therefore, be not straightforward to draw definitive conclusions related to peptide secondary structure motifs. The wild-type peptide DFNKF showed a strong negative peak at 185 nm and positive peaks at 195 nm and 220 nm, which were not present in any of the halogenated peptides. The dibrominated and dichlorinated analogues DF(Br)NKF(Br) and DF(Cl)NKF(Cl), respectively, displayed CD signatures similar to each other with one major negative peak at around 215 nm, consistent with side-chain benzene π-π* transitions,[2] and a minor one at around 240 nm. On the other hand, CD spectra of iodinated peptides DFNKF(I), DF(I)NKF, and DF(I)NKF(I) demonstrated how iodination strongly affects the peptide secondary structure. In particular, a new band at around 200 nm emerged along with a redshift of the band at 240 nm, which in the case of DFNKF(I) became positive. Interestingly, the two strongest gels, those of DF(I)NKF and DF(I)NKF(I), share the most similarities. The reported spectra are consistent with other fibril forming peptides reported in the literature but we hesitate to draw any other firm conclusions about gel structure from CD data.[3]

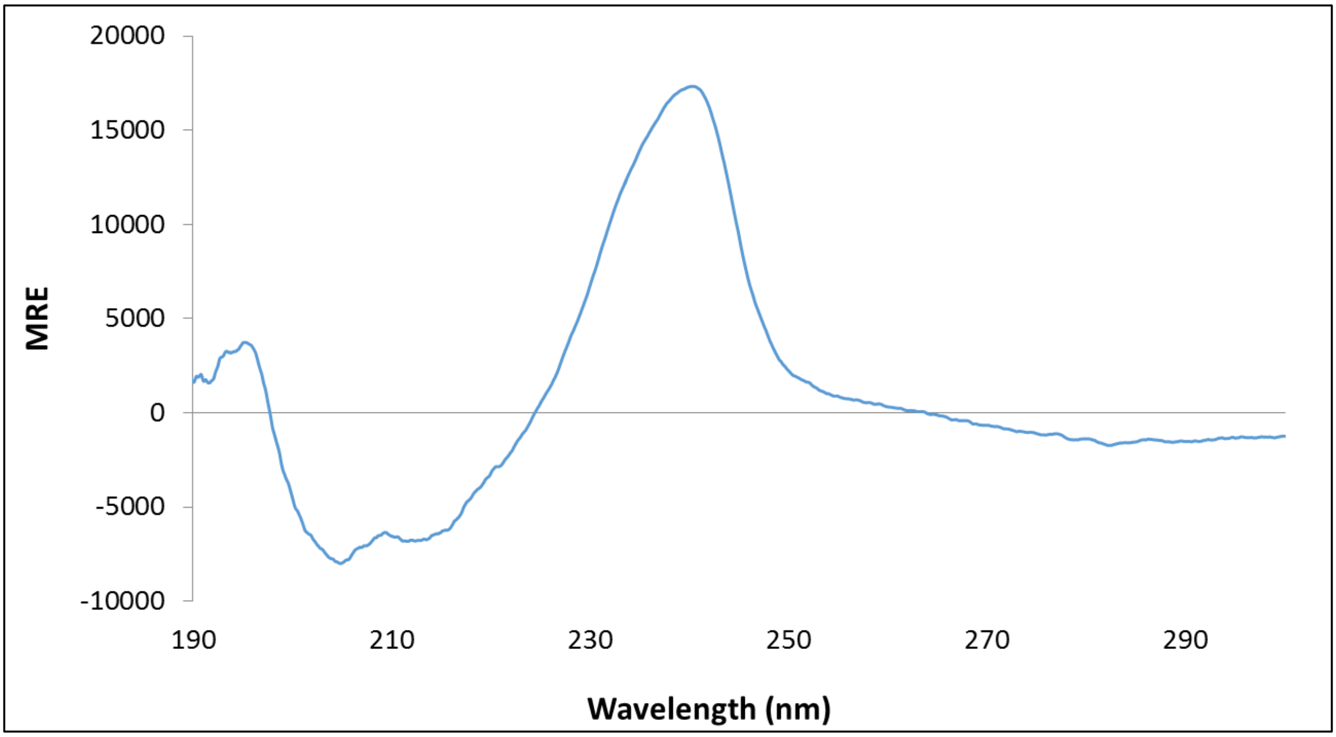

Supplementary Figure 9. Characterisation of the mixed 15 mM DFNKF:1.5 mM DF(I)NKF(I) hydrogel by Circular Dichroism (CD). The obtained CD spectrum of the mixed hydrogel shows the characteristic CD signature of the DF(I)NKF(I) peptide in the 200-220 nm range (negative ellipticity) and the characteristic CD signature of the DFNKF peptide in the 230-250 nm range (positive ellipticity). Interestingly, the global shape of the signal is close to the one obtained with DFNKF(I).

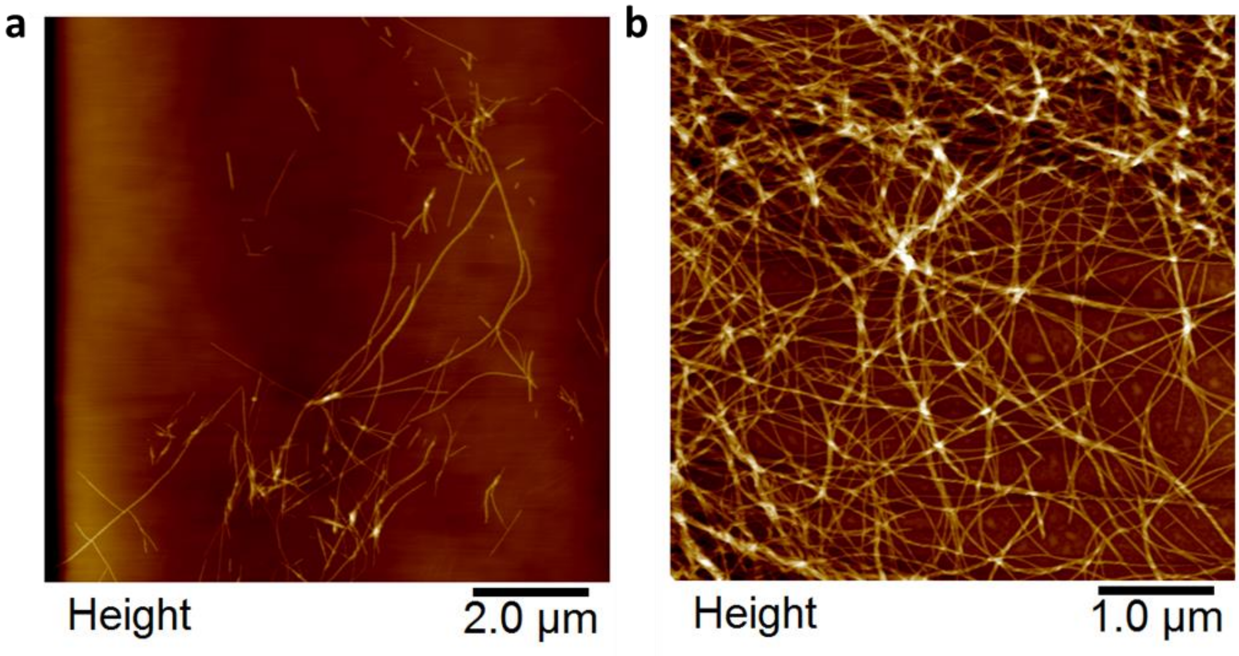

Supplementary Figure 10. Characterisation of mixed hydrogels by AFM. a, Image of mixed hydrogel containing 15 mM DFNKF and 1.5 mM DF(I)NKF(I) showing long fibrils. The image was taken at the interface of the absorbed hydrogel film. **b**, AFM of 15 mM DF(I)NKF(I) showing long entangled fibrils constituting the hydrogel network.

Supplementary Tables

Formula	$C_9H_{12}INO_3$
FW	309.10
Cryst. system	Monoclinic
Space group	$P2_1$
Z	2
a (Å)	6.2312(5)
b (Å)	5.2898(4)
c (Å)	16.4690(13)
β (deg)	96.772(4)
V (Å^3)	539.06(7)
T (K)	103
D_{calc} (g/cm^3)	1.904
μ (mm^{-1})	2.953
Measured reflns	14183
Indep. reflns	4467
R1[onF_0^2,I>2sigma(I)]	0.0368
wR2 (all data)	0.0840
CCDC no.	1049844

Supplementary Table 1. Crystallographic data

Peptide	Minimum Gelation Concentration (mM)	Minimum Gelation Concentration (%w/w)	Time required for gelation
DFNKF	75	5	96h
DFNKF(I)	7.5	0.6	96h
DF(I)NKF	2.5	0.2	168h
DF(I)NKF(I)	5*	0.5	1h
DF(Br)NKF(Br)	8	0.7	3h
DF(Cl)NKF(Cl)	5	0.4	360h

Supplementary Table 2. Minimum gelation concentrations of the studied peptides. It was possible to form a hydrogel with DF(I)NKF(I) at concentration lower than 5 mM, however, the gel was heterogeneous in nature and contained fibrous aggregates within the hydrogel matrix (see image below). For this reason the concentration where no precipitation was observed was taken as the minimum gelation concentration.

**Peptide**	**Time required for gelation at 15 mM**
DFNKF	No gel formed after 30 days
DFNKF(I)	12 h
DF(I)NKF	<30 min
DF(I)NKF(I)	<10 min
DF(Br)NKF(Br)	<30 min
DF(Cl)NKF(Cl)	12 h

Supplementary Table 3. Average gelation times for all of the studied peptides at 15 mM concentration.

Peptide	pH 1.0	pH 2.0	pH 2.8	pH 3.7	pH 5.7	pH 7.5	pH 8.3	pH 9.9	pH 12.7
**DFNKF**									
**DFNKF(I)**			< 5min	< 5min	< 5min	< 5min	< 5min		
**DF(I)NKF**		3h	< 5min	< 5min	< 5min	< 5min	< 5min		
**DF(I)NKF(I)**	20min	< 5min	< 5min	< 5min	< 5min	< 5min	< 5min	30min	
**DF(Br)NKF(Br)**		90min	< 5min	< 5min	< 5min	< 5min	< 5min		
**DF(Cl)NKF(Cl)**		48h	< 5min	< 5min	< 5min	< 5min	< 5min		

gel «milky» gel precipitate solution

Supplementary Table 4. Gelation experiments at different pHs. 10 mM solutions of all halogenated peptides quickly formed gels at the pH range 2.8-8.3. The strongest gelator DF(I)NKF(I) formed a gel in the range 1.0-9.9, showing similar gelation times at these extreme pHs. Peptides were directly weighted in 2mL glass vials and diluted in buffers or in deionized water (18 MΩ.cm). The vials were subsequently sealed, heated up for 1 min at 130 °C, and then slowly cooled down at room temperature. Gelation was determined by the tube inversion method and reported times were taken only when the entire solution became a gel. The aspect of the gel is described following this nomenclature: solution, precipitate (a solution with non-soluble peptides or aggregates), "milky" gel (i.e., forming an heterogeneous gel including solid parts inside, aggregates), gel (a transparent or lightly opaque and stable hydrogel). The actual pH of the gels (or solutions) is measured after 24 hours using a Crison® 5209 micro-electrode. Buffers were used without modification, except for experiments with buffer pH 8 for which drops of 0.1M NaOH solutions were added to fix the pH at 7.5. Before stating that a mixture is not a gel but a solution, the sealed vials are checked during one month, at least.

Peptide	5000 mM	500 mM	100 mM	10 mM	1 mM	0 mM
DFNKF	50 (10)	-	-	-	-	-
DFNKF(I)	5 (2)	195 (25)	245 (35)	1620 (120)	5880 (600)	23040 (2880)
DF(I)NKF	5 (2)	7 (3)	15 (4)	135 (10)	150 (20)	205 (20)
DF(I)NKF(I)	2 (1)	3 (1)	3 (1)	2 (1)	2 (1)	3 (1)
DF(Br)NKF(Br)	2 (1)	5 (2)	15 (3)	30 (5)	65 (10)	130 (20)
DF(Cl)NKF(Cl)	2 (1)	360 (30)	420 (40)	1200 (120)	1740 (240)	1800 (240)

gel «milky» gel solution

Supplementary Table 5. Gelation experiments at different ionic strength. The gelation times and the related standard deviations (in brackets) reported in the table are in min. For these experiments, 5 mM Peptide solutions were used upon increasing molarity of NaCl. Gelation was determined by the tube inversion method and reported times are in minutes (standard deviations in brackets) and taken only when the entire solution became a gel. The aspect of the gel is described following this nomenclature: solution, "milky" gel (i.e., forming an heterogeneous gel including solid parts inside, aggregates), gel (a transparent or lightly opaque and stable hydrogel). Following the procedure described above for the pH-dependent gelation, hydrogels were prepared at 5 mM of starting peptides, with different concentrations of sodium chloride. Stock solutions of NaCl were prepared at 5000 mM, 500 mM, 100 mM, 10mM, and 1mM in deionized water (18.2 MΩ.cm). The ionic strength was calculated using the formula:

$$\mu = \frac{1}{2}\sum_{i=1}^{n} c_i Z_i^2 \qquad (2)$$

Where *i* are all ions present in solution (Na^+ and Cl^-), c_i is the molar concentration of the ion *i*, and Z_i the net charge of the ion *i*.

compound	Ret. Time (std dev.)
DFNKF	15,77 (0,65) min
DFNKF(I)	17,22 (0,61) min
DF(I)NKF	17,51 (0,32) min
DF(I)NKF(I)	20,52 (0,93) min
DF(Br)NKF(Br)	19,92 (0,99) min
DF(Cl)NKF(Cl)	18,52 (0,64) min

Supplementary Table 6. Retention times of studied peptides in reverse-phase HPLC. A gross estimation of the relative hydrophobicity of the studied peptides can be obtained by comparing their retention times in reverse-phase HPLC purification experiments. The most retained peptide is the most hydrophobic one.

Supplementary References

1. Correa, D. H. A. & Ramos, C. H. I. The use of circular dichroism spectroscopy to study protein folding, form and function. *African J. Biochem. Res.* **3**, 164-173 (2009).

2. Smith, A. M *et al.* Fmoc-diphenylalanine self assembles to a hydrogel via a novel architecture based on π–π interlocked β-sheets. *Adv. Mater.* **20**, 37-41 (2008).

3. Cheng, G., Castelletto, V., Jones, R. R., Connona, C. J. & Hamley, I. W. Hydrogelation of self-assembling RGD-based peptides. *Soft Matter* **7**, 1326-1333 (2011).
