# Supplementary material for: Supramolecular amplification of amyloid self-assembly by iodination
Source: Nat Commun. 2015 Jun 30;6:7574. doi: 10.1038/ncomms8574 (PMC4491812; doi:10.1038/ncomms8574)

checkCIF/PLATON report

You have not supplied any structure factors. As a result the full set of tests cannot be run.

THIS REPORT IS FOR GUIDANCE ONLY. IF USED AS PART OF A REVIEW PROCEDURE FOR PUBLICATION, IT SHOULD NOT REPLACE THE EXPERTISE OF AN EXPERIENCED CRYSTALLOGRAPHIC REFEREE.

No syntax errors found. CIF dictionary Interpreting this report

Datablock: luc39

```
Bond precision:  C-C = 0.0051 A          Wavelength=0.71073

Cell:            a=6.2312(5)    b=5.2898(4)        c=16.4690(13)
                 alpha=90       beta=96.772(4)     gamma=90
Temperature:     103 K
```

	Calculated	Reported
Volume	539.06(7)	539.06(7)
Space group	P 21	P 21
Hall group	P 2yb	P 2yb
Moiety formula	C9 H10 I N O2, H2 O	C9 H10 I N O2, H2 O
Sum formula	C9 H12 I N O3	C9 H12 I N O3
Mr	309.10	309.10
Dx,g cm-3	1.904	1.904
Z	2	2
Mu (mm-1)	2.953	2.953
F000	300.0	300.0
F000’	299.18	
h,k,lmax	10,8,27	9,8,26
Nref	4972[2701]	4466
Tmin,Tmax	0.753,0.863	0.438,0.577
Tmin’	0.345	

```
Correction method= # Reported T Limits: Tmin=0.438 Tmax=0.577
AbsCorr = MULTI-SCAN

Data completeness= 1.65/0.90      Theta(max)= 35.650

R(reflections)= 0.0368( 4109)     wR2(reflections)= 0.0840( 4466)

S = 1.111                   Npar= 144
```

The following ALERTS were generated. Each ALERT has the format
test-name_ALERT_alert-type_alert-level.
Click on the hyperlinks for more details of the test.

Alert level C

```
DIFMX01_ALERT_2_C  The maximum difference density is > 0.1*ZMAX*0.75
            _refine_diff_density_max given =      4.454
            Test value =      3.975
DIFMX02_ALERT_1_C  The maximum difference density is > 0.1*ZMAX*0.75
            The relevant atom site should be identified.
PLAT094_ALERT_2_C Ratio of Maximum / Minimum Residual Density ....       2.63 Report
PLAT097_ALERT_2_C Large Reported Max.  (Positive) Residual Density       4.45 eA-3
```

Alert level G

```
PLAT002_ALERT_2_G Number of Distance or Angle Restraints on AtSite          8 Note
PLAT172_ALERT_4_G The CIF-Embedded .res File Contains DFIX Records          1 Report
PLAT176_ALERT_4_G The CIF-Embedded .res File Contains SADI Records          3 Report
PLAT791_ALERT_4_G The Model has Chirality at C8      (Chiral SPGR)          S Verify
PLAT860_ALERT_3_G Number of Least-Squares Restraints .............         12 Note
PLAT899_ALERT_4_G SHELXL97   is Deprecated and Succeeded by SHELXL       2014 Note
```

```
   0 ALERT level A = Most likely a serious problem - resolve or explain
   0 ALERT level B = A potentially serious problem, consider carefully
   4 ALERT level C = Check. Ensure it is not caused by an omission or oversight
   6 ALERT level G = General information/check it is not something unexpected

   1 ALERT type 1 CIF construction/syntax error, inconsistent or missing data
   4 ALERT type 2 Indicator that the structure model may be wrong or deficient
   1 ALERT type 3 Indicator that the structure quality may be low
   4 ALERT type 4 Improvement, methodology, query or suggestion
   0 ALERT type 5 Informative message, check
```

Validation response form

Please find below a validation response form (VRF) that can be filled in and pasted into your CIF.

```
# start Validation Reply Form
_vrf_DIFMX01_luc39
;
PROBLEM: The maximum difference density is > 0.1*ZMAX*0.75
RESPONSE: The crystal quality was very poor and the overall quality of the was quite poor too.
The residual electron density is located on the iodine atom.
;
_vrf_DIFMX02_luc39
;
PROBLEM: The maximum difference density is > 0.1*ZMAX*0.75
RESPONSE: The crystal quality was very poor and the overall quality of the was quite poor too.
The residual electron density is located on the iodine atom.
;
_vrf_PLAT094_luc39
;
PROBLEM: Ratio of Maximum / Minimum Residual Density ....       2.63 Report
RESPONSE: The crystal quality was very poor and the overall quality of the was quite poor too.
The residual electron density is located on the iodine atom.
;
_vrf_PLAT097_luc39
;
PROBLEM: Large Reported Max.  (Positive) Residual Density       4.45 eA-3
RESPONSE:The crystal quality was very poor and the overall quality of the was quite poor too.
The residual electron density is located on the iodine atom.
;
# end Validation Reply Form
```

It is advisable to attempt to resolve as many as possible of the alerts in all categories. Often the minor alerts point to easily fixed oversights, errors and omissions in your CIF or refinement strategy, so attention to these fine details can be worthwhile. In order to resolve some of the more serious problems it may be necessary to carry out additional measurements or structure refinements. However, the purpose of your study may justify the reported deviations and the more serious of these should normally be commented upon in the discussion or experimental section of a paper or in the "special_details" fields of the CIF. checkCIF was carefully designed to identify outliers and unusual parameters, but every test has its limitations and alerts that are not important in a particular case may appear. Conversely, the absence of alerts does not guarantee there are no aspects of the results needing attention. It is up to the individual to critically assess their own results and, if necessary, seek expert advice.

Publication of your CIF in IUCr journals

A basic structural check has been run on your CIF. These basic checks will be run on all CIFs submitted for publication in IUCr journals (*Acta Crystallographica*, *Journal of Applied Crystallography*, *Journal of Synchrotron Radiation*); however, if you intend to submit to *Acta Crystallographica Section C* or *E*, you should make sure that full publication checks are run on the final version of your CIF prior to submission.

Publication of your CIF in other journals

Please refer to the *Notes for Authors* of the relevant journal for any special instructions relating to CIF submission.

PLATON version of 21/04/2015; check.def file version of 09/03/2015

Datablock luc39 - ellipsoid plot

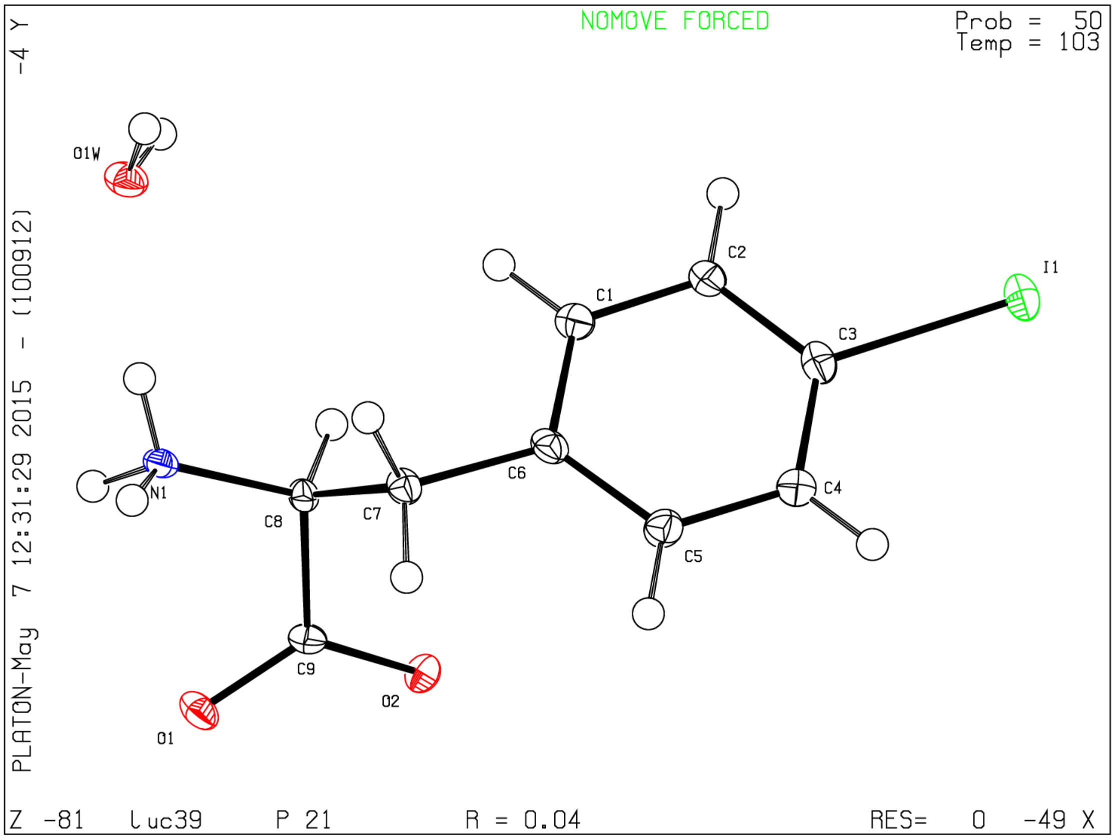

Supplement: Supplementary Data 2 — Check CIF report for p-iodo-phenylalanine CIF [file ncomms8574-s3.pdf]
